# Characterization of the Key Aroma Compounds in Dong Ding Oolong Tea by Application of the Sensomics Approach

**DOI:** 10.3390/foods12173158

**Published:** 2023-08-22

**Authors:** Daoliang Wang, Cainan Wang, Weiying Su, Chih-Cheng Lin, Wei Liu, Yuan Liu, Li Ni, Zhibin Liu

**Affiliations:** 1 Institute of Food Science and Technology, Fuzhou University, Fuzhou 350108, China; 230416027@fzu.edu.cn (D.W.); wangcn@fzu.edu.cn (C.W.); 220416011@fzu.edu.cn (W.S.); nili@fzu.edu.cn (L.N.); 2 Fujian Institute of Food Science and Technology, Fuzhou 350108, China; 3 Department of Biotechnology and Pharmaceutical Technology, Yuanpei University of Medical Technology, Hsinchu 300150, China; lcc@mail.ypu.edu.tw; 4 Fujian College Association Instrumental Analysis Center of Fuzhou University, Fuzhou 350108, China; luv@fzu.edu.cn; 5 Department of Food Science and Technology, School of Agriculture and Biology, Shanghai Jiao Tong University, Shanghai 200240, China; y_liu@sjtu.edu.cn

**Keywords:** Dong Ding oolong tea, key aroma compounds, GC-O-MS, GC × GC-TOF-MS

## Abstract

The Dong Ding oolong tea (DDT), grown and produced in Taiwan, is widely appreciated for its unique flavor. Despite its popularity, research on the aroma components of DDT remains incomplete. To address this gap, this study employed a sensomics approach to comprehensively characterize the key aroma compounds in DDT. Firstly, sensory evaluation showed that DDT had a prominent caramel aroma. Subsequent analysis using gas chromatography-olfactory mass spectrometry (GC-O-MS) and comprehensive two-dimensional gas chromatography time-of-flight mass spectrometry (GC × GC-TOF-MS) identified a total of 23 aroma-active compounds in DDT. Notably, three pyrazine compounds with roasted notes, namely 2-ethyl-5-methylpyrazine, 2-ethyl-3,5-dimethylpyrazine, and 2,3-diethyl-5-methylpyrazine, along with seven floral- and fruit-smelling compounds, namely 6-methyl-5-hepten-2-one, 3,5-octadien-2-one, linalool, (*E*)-linalool oxide, geraniol, (*Z*)-jasmone, and (*E*)-nerolidol, were identified as the key aroma compounds of DDT. Omission experiments further validated the significant contribution of the three pyrazines to the caramel aroma of DDT. Moreover, the content of 2-ethyl-3,5-dimethylpyrazine, 2,3-diethyl-5-methylpyrazine, (*Z*)-jasmone, 6-methyl-5-hepten-2-one and 2-ethyl-5-methylpyrazine was found to be higher in the high-grade samples, while (*E*)-nerolidol, linalool, geraniol and 3,5-octadien-2-one were found to be more abundant in the medium-grade samples. These findings provide valuable information for a better understanding of the flavor attributes of DDT.

## 1. Introduction

**Figure 1:**
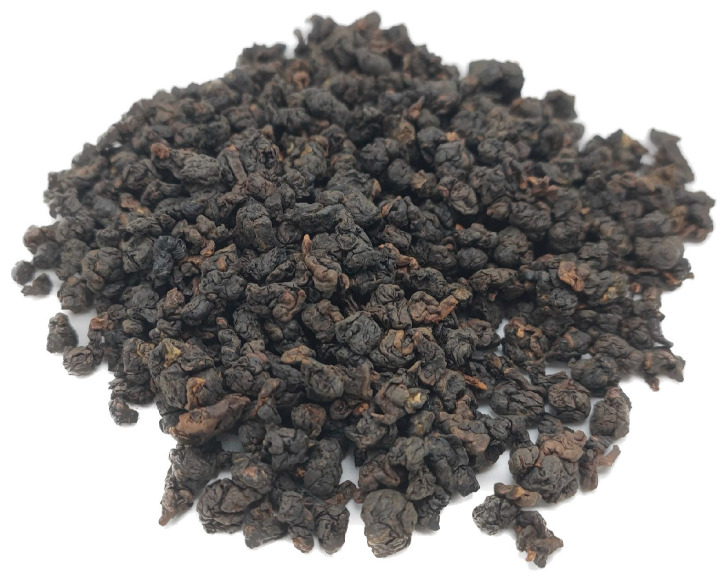
The photo of DDT.

Dong Ding oolong tea (DDT), also known as Tung Ting oolong tea, is a premium oolong tea from Dong Ding Mountain in Taiwan, growing at altitudes ranging from 600 to 1200 m. The mountain is located in the Lugu region of Nantou County in central Taiwan, China, an area long used for growing tea. This region is characterized by fertile soil, cool temperatures, and frequent mists, which facilitate the formation of the distinctive taste and aroma of DDT. The production process of DDT is carried out using experienced tea producers. This process begins with plucking the tea leaves, which are then withered to reduce their moisture content. Following the withering, the leaves are subject to a rolling procedure, which partially breaks the cell walls and initiates the oxidation process. To achieve the desired oxidation level, typically ranging between 15% to 30%, a precise heating step is employed to inactivate enzymes and thus halt the oxidation. The tea leaves are then carefully rolled into the iconic ball shape [1]. Finally, the tea leaves are subjected to a sophisticated, multi-stage roasting process. This process involves low temperatures over a long time, resulting in a mellow, floral, nutty, toasty, caramel, and woody flavor. The finished DDT tea is illustrated in Figure 1.

Owing to its complex and well-balanced flavor profile, coupled with its broad array of health-promoting attributes, Dong Ding oolong tea has garnered global recognition. This fame has led to an increasing demand for this unique tea across the global tea market. However, despite the growing interest in DDT, the comprehensive elucidation of its volatile composition and key aroma components using state-of-the-art analytical techniques remains relatively limited. It was reported that the flavor of medium-fermented DDT lay between that of green tea and black tea [2]. Furthermore, Zhu et al. [3] found that compared with Tieguanyin tea and Dahongpao tea, DDT exhibited a more prominent green and grassy aroma, which may be due to the high content of indole and nerolidol. Nonetheless, beyond the green and grassy aroma, the compounds responsible for floral, nutty, toasty, and caramel flavor remain largely unknown, leaving a notable knowledge gap. Identifying and understanding the key aroma components in DDT helps tea producers optimize their production processes, enhancing the formation of desirable aroma components. Additionally, a comprehensive exploration of the key aroma compounds present in DDT can facilitate the discrimination between DDT products of diverse quality levels.

Using sophisticated analytical techniques, key aroma compounds in tea can be comprehensively characterized, and volatile compounds in complex substrates can be effectively detected and identified. In recent years, advances in analytical techniques have led to the emergence of a novel approach known as “sensomics” [4]. This is a powerful tool that integrates various analytical methods, including gas chromatography, mass spectrometry, and sensory evaluation, offering the possibility of a comprehensive understanding of the aromatic profile of complex food matrices. By combining analytical data with human sensory perception, sensomics provides an in-depth investigation into the flavor and aroma of food and beverage products, such as various types of tea. For example, Huang et al. [5] employed a sensomics approach to identify the aroma-active compounds of Keemun black tea with varying withering degrees. They also found that sun withering yielded elevated concentrations of two aroma-active compounds, namely, linalool and geraniol, while methional played a more significant role in contributing to the aroma profile during warm-air withering. Similarly, by utilizing a sensomics approach, Wang et al. [6] verified the significant contribution of 6-methyl-5-hepten-2-one and acetyl pyrazine as key volatile compounds for the caramel-like and roasty aroma of Wuyi Rock tea. However, it is worth noting that the identification of key aroma compounds in DDT via the sensomics approach remains relatively scarce.

The aim of this study was to comprehensively elucidate the key aroma compounds responsible for the characteristic aroma of Dong Ding oolong tea, utilizing the sensomics approach. To achieve this, a total of 27 DDT samples were collected from various locations within the Dong Ding mountain region. Firstly, a panel of five tea evaluation experts evaluated the sensory attributes of 27 DDT samples. Subsequently, the key aroma compounds of DDT were identified and validated using the implementation of gas chromatography-olfactory mass spectrometry (GC-O-MS), comprehensive two-dimensional gas chromatography time-of-flight mass spectrometry (GC × GC-TOF-MS), and aroma recombination and omission experiments. Finally, a comparative analysis of the key aroma compounds present in varying grades of DDT was performed employing multivariate statistics.

## 2. Materials and Methods

### 2.1. Chemicals

Aroma compound standards were obtained commercially and used for the quantification of the following volatiles: 6-methyl-5-hepten-2-one, 2-acetylfuran, caproicacidhexneylester, and 2-acetylpyrrole (TMstandard, Changzhou, China); 2-ethyl-3,5-dimethylpyrazine, furfural, linalool, (*E*,*E*)-2,4-heptadienal, 5-methylfurfural, methyl salicylate, 1-furfurylpyrrole, geraniol, and phenylethyl alcohol (Macklin Biochem, Shanghai, China); 2,3-diethyl-5-methylpyrazine (TCI, Shanghai, China); 3-hexen-1-ol, 2-ethyl-5-methylpyrazine and (*E*)-linalool oxide (CATO, Eugene, OR, USA); 2-methyl-4-methoxyaniline and 3,5-octadien-2-one (ChemService Inc., West Chester, PA, USA); (*Z*)-Jasmone (Yuanye Co., Ltd., Shanghai, China); and (*E*)-nerolidol (Bide Pharmatech Co., Ltd., Shanghai, China). The purity of all aroma compound standards used in this study is over 95%.

The standard of 2-octanol (Dr. Ehrenstorfer, Augsburg, GER) was gained to semi-quantify volatiles. A standard of N-alkanes mixed (C_7_–C_40_) (O2SI, Charleston, SC, USA) was purchased for the identification of aroma compounds. Sodium chloride (NaCl, analytically pure, Sinopharm Chemical Co., Ltd., Shanghai, China) was used for HS-SPME.

### 2.2. DDT Samples

A total of 27 DDT samples were collected in Taiwan (China). The information on all DDT samples is presented in Table S1.

### 2.3. Evaluation of Sensory Attributes

The sensory attributes of DDT samples followed the guidelines of the Chinese national standards for sensory evaluation of oolong tea (GB/T 23776-2018) [7]. In brief, 5 g of DDT sample was accurately weighed and placed in a 110 mL tea bowl. Boiling deionized water was added to the bowl, and the tea was allowed to steep for 2 min. Subsequently, the tea infusion was carefully poured into an empty tea bowl. The process of brewing was repeated for two additional rounds with the same method using the remaining tea leaves. After completing the three rounds of brewing, the sensory panel members conducted the sensory evaluation of all the tea infusions. The results of the sensory evaluation were then recorded.

### 2.4. GC-O-MS Analysis

Prior to GC-O-MS analysis, the volatiles were extracted from all samples of DDT using the previously established HS-SPME method [8]. In brief, 1.5 g of NaCl and 0.1 g of powdered DDT sample were added to an 18 mL headspace bottle and then added to 10 μL 2-octanol (10 mg/L) in the bottle serving as the internal standard. After that, boiling deionized water (5 mL) was added to brew the DDT sample and incubated in a water bath at 50 °C for 15 min. Finally, SPME fibers were inserted and extracted at 50 °C for 50 min.

After extraction, the volatile compounds in the sample were subjected to GC-O-MS analysis using the method previously reported [9,10]. Specifically, a 7890A gas chromatograph (Agilent, Santa Clara, CA, USA) equipped with an HP-INNOWAX column (30 m × 250 μm × 0.25 μm, Agilent, USA) was coupled to a 5975C mass spectrometer (Agilent, USA) for the purpose of separation and identification. The GC-MS instrument was coupled to an olfactory detector port (HCU-15, Brechbuhler, Switzerland) to form a GC-O-MS system. The GC injection port temperature was set at 250 °C. High purity He (purity > 99.999%, 1 mL/min) was employed as the carrier gas. The temperature program was set as follows. Firstly, the oven temperature was held at 40 °C for 5 min, and then it was heated up to 120 °C (at a rate of 3 °C/min). The temperature was further raised to 240 °C (at a rate of 6 °C/min) and maintained for 5 min. Finally, the post-run procedure was carried out at 240 °C for 5 min. The electron hit was performed with an energy of 70 eV. The mass spectrum scanning ranged from 35 to 450 amu. A NIST11.L standard spectrum library was used to compare with the obtained results, and each compound was characterized using the normal alkane retention index (RI) of C_7_–C_40_. 2-octanol was used as an internal standard for semi-quantitative analysis of phenethyl benzoate.

Subsequently, ADA analysis was performed using the previously reported methods [9]. A sensory panel consisting of three trained non-smokers from the Institute of Food Science and Technology at Fuzhou University identified potential aroma-active compounds in DDT samples. The panelists recorded the beginning and ending times of the perceived aroma, sensory attributes, and intensity of the aroma. The ADA analysis was independently conducted in triplicate by each panelist.

### 2.5. GC × GC-TOF-MS Analysis of the Volatile Compounds

GC × GC-TOF-MS analysis was performed using a 7890B GC (Agilent, USA) coupled with a Hexin EI-TOF MS 0620 (Hexin, Guangzhou, China), following a previously reported method with slight modifications [11]. The first GC column was HP-INNOWAX polar column (30 m × 250 μm × 0.25 μm, Agilent, USA), and the second GC column was Rxi-17 Sil MS medium polar column (2 m × 250 μm × 0.25 μm, Restek, PA, USA). The injection port temperature was set at 250 °C, and high purity He (99.999%, 1.4 mL/min) was used as the carrier gas. The temperature program of the first column oven was consistent with those used in GC-MS as aforementioned, whereas the temperature of the second column oven was maintained 5 °C higher than that of the 1D oven. The 2D modulation period was set to 6 s. For MS detection, the scanning rate was 101 spectra/s, the EM voltage was set to 70 eV, the EI ion source temperature was set to 250 °C, the solvent delay time was set to 300 s, and the MS was scanned in the range of 45–550 amu.

### 2.6. Quantitative Analysis of Potential Odorants in DDT

Following the identification of the aroma-active compounds in DDT samples, external standard curves for these aroma-active compounds were established using GC × GC-TOF-MS for quantitative analysis. The correlation coefficient of calibration curves for all external standards was between 0.9905 and 0.9995, and the concentration ranged from 1.0 to 200.0 mg/L. Subsequently, the actual concentration of aroma-active compounds in the samples was calculated using the corresponding linear equations. In addition, the odor activity values (OAV) of each aroma-active compound were calculated according to the method reported in the literature [12], wherein OAV = C/OT (“C” represents the concentration of the compound in DDT tea infusion and “OT” represents the threshold concentration of the compound in water). Additionally, FD is defined as the maximum dilution of a compound at which its aroma can still be perceived. In general, volatiles with both FD and OAV values ≥ 1 are considered significant contributors to the overall aroma of DDT and thus were identified as key aroma compounds. The contribution of these key aroma compounds was then validated using aroma recombination and omission experiments.

### 2.7. Aroma Recombination and Omission Experiments

Using previously reported methods, with some modifications [9]. Firstly, 100 g DDT was brewed three times with 5000 mL of boiling deionized water. Subsequently, the tea residue was dried in an oven at 60 °C to form a blank tea matrix. Additionally, all key aroma compounds were added to the water according to their actual concentrations in DDT tea infusion. Finally, the blank tea matrix was added to this solution to form the recombinant model.

A sensory panel consisting of 10 trained non-smoke experts (6 females and 4 males, aged 22–26 years) from the Institute of Food Science and Technology at Fuzhou University performed a comparative evaluation of the tea infusion and recombinant models. Sensory evaluation was carried out in accordance with previously reported procedures [9]. Prior to the evaluation, the panelists reached a consensus on the aroma attributes of DDT, which included caramel, woody, nutty, fatty, green, fruity, floral, and roasted. Subsequently, the panelists evaluated the tea infusion and recombinant models on a six-point scale for each of the eight aroma attributes.

The omission experiment was carried out in accordance with the methods reported in previous studies [9]. In brief, the omitted model is prepared by removing specific individual compounds from the complete recombinant model. Panelists were forced to choose a unique single model between the omitted model and two unabridged recombinant models. All experiments were conducted in triplicate.

### 2.8. Statistical Analysis

GC × GC-TOF-MS data were integrated by Hexin Canvas (Version 1.01.00.0). Multivariate statistical analyses, including heatmap plots, PCA, and HCA, were conducted using R software (Version 4.2.0). GraphPad Prism software (Version 8.0.26) was used for graphing. All determinations were performed in triplicate, and data were expressed as mean ± SD.

## 3. Results and Discussion

### 3.1. Odor Attributes of DDT

A panel of five tea evaluation experts performed odor attribute descriptions and ratings on 27 samples of DDT. Based on the rating, all tea samples were categorized into three grades: high, medium, and low, as shown in Table S2. Among these, 9 samples were rated as high-grade, 9 as medium-grade, and 9 as low-grade. The experts associated floral, caramel, and fresh aromas with the high-grade tea samples while noting the presence of unpleasant odors such as “thick” and “pungent” in the low-grade samples. In addition, the experts observed a caramel-like aroma in one-third of the DDT samples (9 out of 27 samples), suggesting that caramel aroma may contribute significantly to DDT’s signature aroma. In order to further elucidate the key aroma compounds of DDT, its volatile fingerprints were subjected to comprehensive analysis.

### 3.2. Identification of Aroma-Active Compounds by Using GC-O-MS Combined with ADA

**Figure 2:**
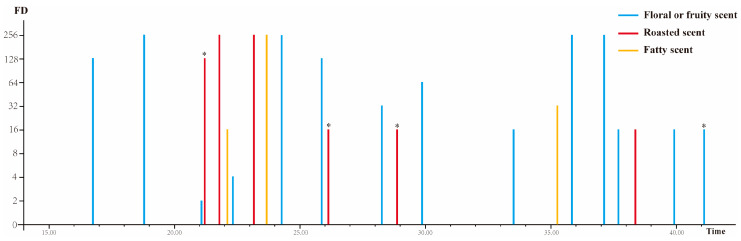
Flavor dilution chromatograms of the aroma-active regions identified in DDT. Different colors indicate the different aroma types. The symbol “*” indicates the compounds are unidentified in GC-O-MS and are subject to further identification using GC × GC-TOF-MS.

To identify the aroma-active compounds potentially contributing to the aroma of DDT, the volatiles extracted by using HS-SPME and its serial dilutions were analyzed by GC-O-MS combined with the ADA approach. The panelists identified 23 aroma-active regions along the GC chromatography (Figure 2). Among them, 3-hexen-1-ol (green, leafy), 2-ethyl-3,5-dimethylpyrazine (peanut, nutty, caramel), 2-acetylfuran (sweet, balsamic, almond), 2,3-diethyl-5-methyl-pyrazine (musty, nut, meaty), geraniol (sweet, floral, fruity), and phenylethyl alcohol (floral, rose) reached the maximum value set in the experiment. Lan et al. [9] have previously reported an increase in pyrazine and furan compounds in Qingxin Oolong tea after roasting. These compounds were also detected in Wuyi Rock tea, another type of roasted oolong tea, suggesting their generation during the tea roasting process [8]. These aroma-active compounds with high FD values were commonly associated with roasting, caramel, and sweet flavors, which aligned well with the results of sensory evaluation.

Notably, four compounds were classified as “unidentified compounds” and were marked with “*” in Figure 2. Although detectable by the panelists, the identification based on mass spectrometry data was inconclusive due to the co-elution in GC separation. Their aroma attributes and FD values are presented in Table 1. Among them, unidentified compounds **1**, **2**, and **3** showed coffee and cocoa flavors, while unidentified compound **4** showed rose and honey flavors.

### 3.3. Identification of “Unidentified” Aroma-Active Compounds by Using GC × GC-TOF-MS

Using GC × GC-TOF-MS, the compounds with the same retention time in the first column can be further separated on the second column, resulting in improved separation efficiency. In this study, we employed a two-dimensional GC system consisting of the HP-INNOWAX polar chromatography column and the Rxi-17 Sil MS medium polar chromatography column. This choice was based on previous reports suggesting that the polar column × medium polar column combination is more effective for chromatography separation, particularly for polar compounds, than the combinations of polar column × non-polar column and non-polar column × polar column [13]. In addition, the modulation period also has an important effect on the separation efficiency [14]. After optimization, we found that a modulation period of 6 s allowed for an even distribution of the compounds in the chromatogram center.

**Figure 3:**
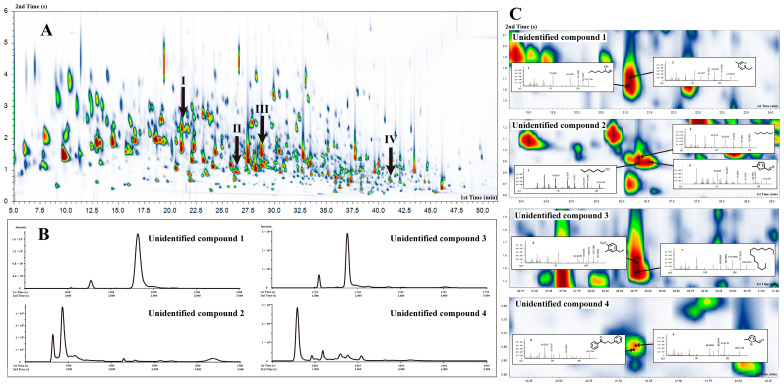
GC × GC-TOF-MS 2D topographic maps of DDT (**A**) and the chromatograms of the four unidentified compounds in the second column (**B**). Zoom in the regions of the four unidentified compounds and the detected mass spectrum fragmentations (**C**).

In the GC × GC-TOF-MS chromatogram, it was evident that multiple compounds experienced co-elution in the first dimension GC, making their accurate identification challenging. However, the introduction of a second column significantly improved the identification process. As shown in Figure 3B, four unidentified compounds were efficiently separated in this two-dimensional system.

For the identification of compounds, we first identified peaks with a signal-to-noise ratio of ≥100 using a deconvolution procedure based on mass spectrum differences. For the four unidentified compounds, further manual deconvolution was performed. As shown in Figure 3C, taking unidentified compound **2** as an example, we observed that five compounds co-eluted in the first column but effectively separated in the second column. The automatic program identified two of the compounds as (*3E*)-1-methoxy-3-hexene and methoxyacetic acid-3-tridecyl ester. However, the remaining three compounds were still difficult to identify. Therefore, the detected mass spectrum ion fragments were compared with the Spectral Database for Organic Compounds to achieve manual identification. In addition, we compared the RI obtained in this study with the reported data to further validate our results [15,16,17]. Consequently, these three compounds were identified as hexan-1-ol, heptane, and 5-methylfurfural. Considering that panelists described the aroma of this region as coffee and roasted aroma in the GC-O-MS analysis, only 5-methylfurfural matched this specific aroma profile. Furthermore, since 5-methylfurfural exhibited the highest peak intensity among the five co-elution compounds, it can be concluded that unidentified compound **2** was, indeed, 5-methylfurfural. With this approach, all four unidentified compounds were accurately identified. The results and their corresponding aroma characteristics, as reported by panelists, are shown in Table 1.

### 3.4. Identification of Key Aroma Compounds of DDT by OAV

ADA serves as a preliminary approach for identifying potential aroma-contributing compounds in tea; however, the effect of the matrix on aroma release and perception may be overlooked [18]. In addition, solely considering the concentration of a compound does not necessarily determine its significant contribution to the overall flavor of DDT. Thus, it must be paired with its threshold in the water. For this reason, a more effective assessment of individual compounds’ contribution to tea aroma can be achieved via OAV calculations. With the exception of phenethyl benzoate, which was semi-quantified using 2-octanol, the actual concentration of the remaining 22 compounds was determined using their corresponding external standard curves (Table S3). Next, the OAV values of the 23 compounds were obtained. It is generally believed that compounds with OAV > 1 contribute more significantly to DDT aroma, while compounds with OAV < 1 contribute to a lesser extent [19]. Table 2 presents comprehensive information for the 23 aroma-active compounds, including names, 1D and 2D retention times, RI values, odor descriptions, odor thresholds (in water), content in tea infusion, and OAV values. Odor descriptions and odor thresholds were derived from FEMA databases or published literature [19,20,21]. Notably, 10 compounds exhibited OAV values > 1, with 2,3-diethyl-5-methylpyrazine having the highest OAV (1406.29), followed by linalool (136.51) and 3,5-octadien-2-one (110.84). The chemical structures of these ten compounds are demonstrated in Supplemental Material Figure S1. These compounds with high OAV may contributed significantly to the aroma of DDT. In addition, three pyrazine compounds may contribute significantly to the caramel aroma of DDT. These results were further verified via aroma recombination and omission experiments.

### 3.5. Aroma Recombination and Omission Experiments

**Figure 4:**
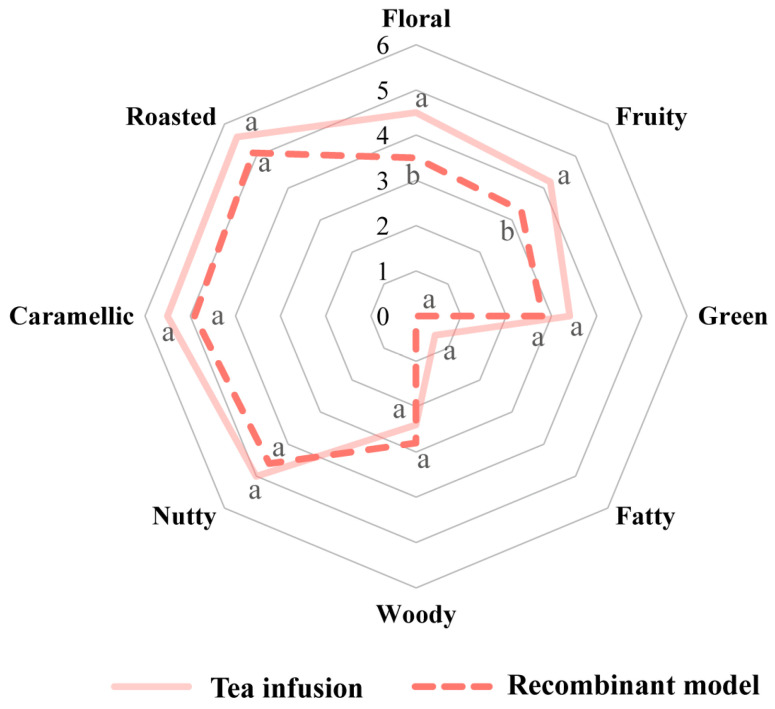
Aroma attributes of the tea infusion of DDT (solid line) and its corresponding aroma recombinant (dashed line), “a” and “b” represent significantly different concentrations (*p* < 0.05).

Aroma recombination experiments are widely used to verify the contribution of specific compounds to the overall flavor of foods [22]. The recombinant model in this study was constructed based on the actual contents of the 10 key aroma compounds with OAV ≥ 1 in DDT tea infusion. Subsequently, the panelists compared the recombinant model with the actual tea infusion, and the results are shown in Figure 4. It was observed that the recombinant model had a strong similarity with DDT tea infusion. Notable, the scores of roasted, nutty, and caramel aroma in the recombinant model were higher, which was consistent with the aroma attributes of DDT tea infusion. Overall, the characteristics of all key aroma compounds in DDT tea infusion were successfully reproduced in the recombinant model.

Subsequently, omission experiments were performed to gain a deeper understanding of the contribution of individual key aroma compounds to the overall aroma of DDT. A recombinant model of aroma compounds was established, with a single key aroma compound omitted at a time. Next, panelists were forced to choose a unique recombinant model among three recombinants. The omission models of 10 key aroma compounds were found to be significantly different from the complete recombinant model (*p* < 0.05) (Table 3). In addition, omitting 6-methyl-5-hepten-2-one, 2-ethyl-5-methylpyrazine, 2-ethyl-3,5-dimethylpyrazine, (*Z*)-jasmone, and 2,3-diethyl-5-methylpyrazine, respectively, from the recombinant models resulted in highly significant differences (*p* < 0.01). These results suggest that they contribute significantly to the overall aroma of DDT. In order to investigate the contributions of three pyrazines, namely 2-ethyl-5-methylpyrazine, 2-ethyl-3,5-dimethylpyrazine, and 2,3-diethyl-5-methylpyrazine, to the caramel aroma of DDT, we established two recombinant models for comparative analysis. One model solely incorporated the three pyrazines, while the other model omitted them. The results indicated that the absence of caramel aroma was perceived by the panelists in the recombinant model that omitted the three pyrazines, while the difference in caramel aroma was not significant in the recombinant model that omitted other compounds. Therefore, the three pyrazines were determined to contribute significantly to the caramel aroma in DDT.

### 3.6. Comparison of Key Aroma Compounds in DDT with Different Grades

Based on the OAV values and the results of aroma recombination and omission experiments, three caramel aroma compounds, namely, 2-ethyl-3,5-dimethylpyrazine, 2-ethyl-5-methylpyrazine, and 2,3-diethyl-5-methylpyrazine, and seven floral and fruity aroma compounds, namely, 3,5-octadien-2-one, linalool, (*E*)-nerolidol, 6-methyl-5-hepten-2-one, geraniol, (*Z*)-jasmone, and (*E*)-linalool oxide, were determined as the key aroma compounds of DDT.

**Figure 5:**
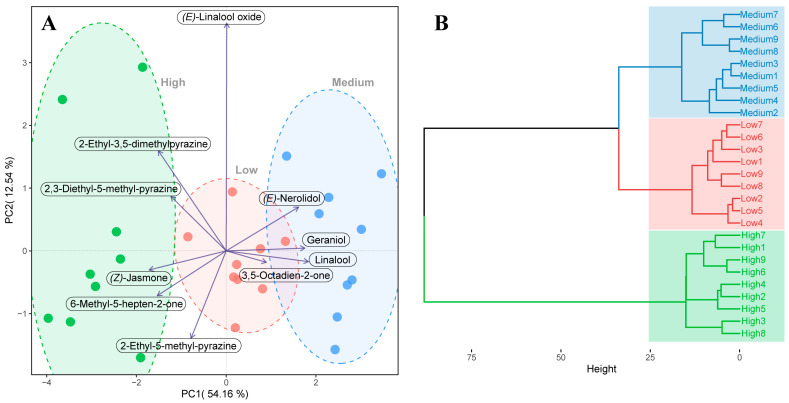
Principal component analysis and PCA loading plot (**A**) and hierarchical cluster analysis (**B**) of the 10 key aroma compounds with OAV > 1 across all DDT samples with three grades, including high grades, medium grades, and low grades.

**Figure 6:**
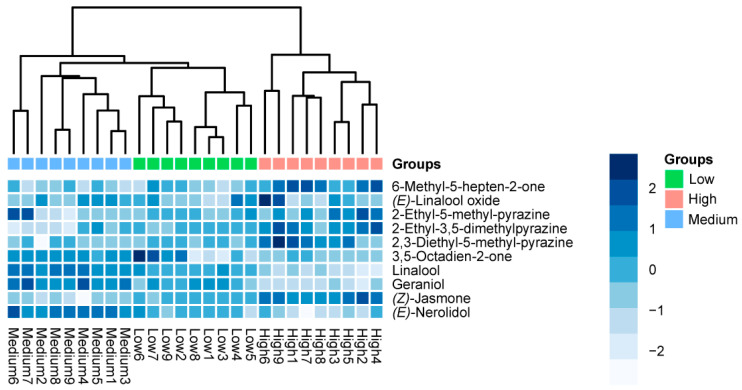
Heat map of the content distribution of the 10 compounds with OAV > 1 across all DDT samples with three grades, including high grades, medium grades, and low grades.

According to the actual content of the 10 key aroma compounds presented in DDT tea infusions, comparative analyses of these compounds among different grades of DDT were conducted using PCA and HCA (Figure 5). The PCA score plot demonstrated an accumulated explanatory variance of 66.70% for the first two principal components, which was enough to explain the information regarding the key aroma compounds across all DDT samples. It is clearly demonstrated that all samples were divided into three different clusters, consistent with their respective grades. The PCA loadings of the 10 compounds were further plotted to pinpoint characteristic key aroma compounds unique to each DDT grade (Figure 5A). Notably, the compounds located on the left, namely 2-ethyl-3,5-dimethylpyrazine, 6-methyl-5-hepten-2-one, (*Z*)-jasmone, 2,3-diethyl-5-methylpyrazine, and 2-ethyl-5-methylpyrazine, were the most significant contributors to the separation of high-grade DDT samples from other samples. Conversely, the compounds located on the right, i.e., (*E*)-nerolidol, linalool, geraniol, and 3,5-octadien-2-one, represented the characteristic key aroma compounds of the medium-grade samples. Next, HCA was conducted to visualize the overall comparison of the key aroma compound profiles (Figure 5B). The dendrograms revealed a clear clustering pattern consistent with their respective grades. The results from both PCA and HCA indicated observable differences in the key aroma compounds among the different grades of DDT. Furthermore, a heatmap plot was employed to provide a visual and comprehensive comparison of the compounds based on their content, as reflected in Figure 6. Notably, five compounds, i.e., 2-ethyl-5-methylpyrazine, 6-methyl-5-hepten-2-one, 2-ethyl-3,5-dimethylpyrazine, (*Z*)-jasmone, and 2,3-diethyl-5-methylpyrazine, were significantly more abundant in high-grade samples. On the other hand, four compounds, i.e., 3,5-octadien-2-one, (*E*)-nerolidol, geraniol, and linalool, were significantly more abundant in medium-grade samples.

Among these key aroma compounds, it was observed that the content of the three pyrazines was notably higher in the high-grade DDT. These pyrazines play a substantial role in imparting the characteristic caramel aroma to DDT. These compounds are considered typical products of the Maillard reaction, generated using thermal reactions involving sugars and amino acids. Zhan et al. [23] demonstrated that in the model thermal reactions of fructose with alanine or lysine, 2,3-diethyl-5-methylpyrazine, and 2-ethyl-3,5-dimethylpyrazine were produced in substantial amounts at temperatures above 110 °C, reaching their maximum at 130 °C. Moreover, the presence of abundant _L_-theanine and d-glucose in tea also contributes notably to the formation of pyrazine compounds [24]. Specifically, Wang et al. [25] found that 2-ethyl-5-methylpyrazine contributes significantly to the high-fired aroma of tea after roasting, and the intense roasting process may promote the production of 2-ethyl-5-methylpyrazine. Considering the intense roasting endured by the high-grade DDT, it is reasonable to predict the extensive presence of these three pyrazines in the high-grade DDT, signifying their contribution to its characteristic caramel aroma.

The floral and fruity aroma compounds, namely, geraniol, linalool, and its oxides belong to the class of monoterpene alcohols, with glycosides serving as their precursors. These compounds are commonly found in various oolong tea varieties, including Wuyi Rock tea [26], Tieguanyin tea [27], and Huangmeigui tea [28]. Geraniol and linalool are released from geranyl pyrophosphate precursors via the action of geraniol synthase and linalool synthase, respectively, after hydrolysis of their glucoside precursors [29]. Liu et al. [10] reported that their content tended to decrease gradually during the roasting process, possibly due to the evaporation, thereby explaining their lower content in the heavily roasted high-grade DDT. The presence of 6-methyl-5-hepten-2-one is attributed to oxidative degradation via the carotenoid pathway. Wang et al. [25] reported that its content increased significantly with the drying temperature increasing, which could be linked to the high temperatures during roasting of high-grade DDT. Although 3,5-octadien-2-one is less abundant, it may evaporate during roasting due to its lower boiling point [30]. However, because of its low odor threshold, it still makes an important contribution to the aroma of DDT. Regarding (*E*)-nerolidol, its formation results from the photooxidative degradation of phytofluene in tea [31]. The continuous mechanical damage that DDT undergoes during withering and drying may accelerate this reaction [32]. Notably, previous studies have indicated that 3,5-octadien-2-one and (*E*)-nerolidol were closely related to the chestnut-like aroma in tea infusion [33]. As for (*Z*)-jasmone, it originates from jasmonic acid via decarboxylation [31]. At high concentration, (*Z*)-jasmone has a pungent odor, while at low concentration, it presents a pleasant jasmine aroma [12]. In this study, the concentration of (*Z*)-jasmone was determined to be in the range of 20.99 to 74.61 μg/L across all DDT infusions. This concentration is anticipated to contribute to a delightful jasmine-like floral aroma, enhancing the overall sensory experience of the tea. Nevertheless, the specific formation mechanism of these compounds during DDT processing requires further exploration and investigation.

## 4. Conclusions

This study comprehensively characterized the sensory attributes and key aroma compounds of DDT. Utilizing GC-O-MS and GC × GC-TOF-MS techniques, a total of 23 aroma-active compounds were identified in DDT. Among these, 10 compounds with OAV > 1 were recognized as the key aroma compounds, playing a significant role in shaping the overall aroma profile of DDT. Their contribution to the overall aroma of DDT was verified using aroma recombination and omission experiments. Notably, the variations in the key aroma compounds among different grades of DDT were also investigated, with pyrazines emerging as the most characteristic compounds in high-grade DDT, imparting the caramel aroma. Further research is warranted to elucidate the pathways by which these compounds are formed during the processing of DDT. Nevertheless, these findings still provided valuable insights to strengthen our understanding of the flavor of this unique oolong tea.

## Figures and Tables

**Table 1:** Co-eluted aroma-active compounds identified by GC × GC-TOF-MS.

NO.	Co-Eluted Compounds	Odor Description	Odor Reported by Panelist	Determined Aroma-Active Compound
1	1-Octen-3-ol	Cucumber, earth, fat	Nutty	2-Ethyl-5-methyl-pyrazine
	2-Ethyl-5-methyl-pyrazine	Coffee, beany, nutty		
	4-(Hydroxymethyl)-3-cyclohepten-1-one	-		
	4-Ethenyl-1,3-benzenediol	-		
2	Hexan-1-ol	Fruity	Coffee, roasted	5-Methylfurfural
	(*3E*)-1-Methoxy-3-hexene	-		
	Heptane	Oil, pungent, sweet		
	5-Methylfurfural	Burnt, roasted, smoky		
	Methoxyacetic acid-3-tridecyl ester	-		
3	2-Methyl-4-methoxyaniline	Coffee, woody	Coffee	2-Methyl-4-methoxyaniline
	Hexanoic acid, hexyl ester	-		
	Hexadecane	Alkane		
4	2-Phenylnitroethane	-	Sweet, honey	Phenethyl benzoate
	Benzoic acid, hexyl ester	Green, waxy, fruity		
	5-Methyl-1H-pyrrole-2-carboxaldehyde	Savory		
	Phenethyl benzoate	Rose, honey, floral		
	Cedrol	Cypress-like		
	2-Furan-2-yl-1H-pyrrole	-		

**Table 2:** The actual concentration, odor description, and OAV of all aroma-active compounds.

NO.	Compounds	RT1 (min)	RT2 (s)	RI	Odor Description ^a^	OT (μg/L) ^b^	OAV	Concentration (μg/L)
1	6-Methyl-5-hepten-2-one	16.73	1.980	1338	Citrus, green, musty	50	6.89	344.21 ± 96.83
2	3-Hexen-1-ol	17.93	0.610	1380	Green, leafy	110	0.03	3.62 ± 1.15
3	(*E*)-Linalool oxide	21.13	2.612	1428	Green, floral	0.32	26.49	8.41 ± 2.81
4	2-Ethyl-5-methylpyrazine	21.14	1.638	1431	Coffee, beany, nutty	16	6.30	100.84 ± 0.42
5	2-Ethyl-3,5-dimethylpyrazine	21.73	2.812	1456	Peanut, nutty, caramellic	0.04	30.81	1.20 ± 0.64
6	Furfural	22.03	1.798	1467	Sweet, woody, almond	770	<0.01	3.42 ± 1.18
7	(*E*,*E*)-2,4-Heptadienal	22.13	2.027	1482	Fatty, green, oily	10,000	<0.01	15.57 ± 10.23
8	2,3-Diethyl-5-methylpyrazine	23.16	2.114	1489	Musty, nut, meaty	0.084	1406.29	118.23 ± 69.20
9	2-Acetylfuran	23.53	0.781	1496	Sweet, balsamic, almond	10,000	<0.01	5.42 ± 2.22
10	3,5-Octadien-2-one	24.40	3.758	1528	Fatty, fruity, mushroom	0.1	110.84	11.01 ± 8.19
11	Linalool	25.93	2.055	1539	Citrus, floral, sweet	0.22	136.51	30.03 ± 7.01
12	5-Methylfurfural	26.34	1.874	1552	Burnt, roasted, smoky	1110	0.01	13.81 ± 6.04
13	Hotrienol	28.23	3.331	1605	Sweet, tropical, floral	110	0.53	58.78 ± 18.02
14	2-Methyl-4-methoxyaniline	28.86	1.501	1613	Coffee, woody	n.f.	-	1.02 ± 0.20
15	Caproicacidhexneylester	29.74	3.331	1656	Fruity, green, waxy	195	0.07	14.41 ± 9.22
16	Methyl salicylate	33.53	2.157	1745	Wintergreen, minty	40	0.04	1.84 ± 0.81
17	1-Furfurylpyrrole	35.13	1.574	1801	Plastic, green, waxy	100	0.33	33.42 ± 9.21
18	Geraniol	35.93	1.775	1830	Sweet, floral, fruity	7.5	59.22	444.20 ± 189.62
19	Phenylethyl alcohol	37.14	0.438	1909	Floral, rose	390	0.02	6.61 ± 3.62
20	(*Z*)-Jasmone	37.83	1.347	1942	Woody, herbal, floral	7	6.84	47.80 ± 26.81
21	2-Acetylpyrrole	38.33	1.270	1959	Musty, nut, skin	58,585.26	<0.01	2.01 ± 1.21
22	(*E*)-Nerolidol	39.93	1.023	1975	Floral, green, citrus	250	1.13	281.21 ± 125.83
23	Phenethyl benzoate	41.06	0.625	2081	Rose, honey, floral	n.f.	-	3.80 ± 1.57

^a^ All the odor descriptions were obtained from the FEMA database or published literature [19,20,21]. ^b^ Odor thresholds were the aroma thresholds in water, which were obtained from the literature [19,20,21], “n.f.” means the odor threshold of the compound was not found.

**Table 3:** Effects of aroma compounds with OAV > 1 on overall aroma profile.

NO.	Omitted Compounds ^a^	n ^b^	Significance ^c^
1	All odor-active compounds	10	***
2	6-Methyl-5-hepten-2-one	8	**
3	(*E*)-Nerolidol	7	*
4	2-Ethyl-5-methylpyrazine	8	**
5	2-Ethyl-3,5-dimethylpyrazine	8	**
6	2,3-Diethyl-5-methylpyrazine	8	**
7	3,5-Octadien-2-one	7	*
8	Linalool	7	*
9	Geraniol	7	*
10	(*Z*)-Jasmone	8	**
11	(*E*)-Linalool oxide	7	*
12	All pyrazine compounds with OAV > 1	10	***
13	All odor-active compounds except pyrazines	3	n.s.

^a^ Panelists assessed the absence of caramel aroma for models 12 and 13. ^b^ In the triangle test, the number of correct judgments of 10 panelists. ^c^ If the exclusion of a particular compound leads to a perceivable alteration in the overall aroma of the recombinant model, a “*”, “**”, or ”***” symbol will be assigned. Significance: *, *p* < 0.05; **, *p* < 0.01. ***, *p* < 0.001; “n.s.”, non-significant.

## Data Availability

The data used to support the findings of this study can be made available by the corresponding author upon request.

## Supplementary Materials

The following supporting information can be downloaded at: https://www.mdpi.com/article/10.3390/foods12173158/s1, Table S1: Tea samples information; Table S2: Sensory scores and sensory grades of DDT; Table S3: Standard curve of aroma-active compounds of DDT; Figure S1: Chemical structure of 10 key aroma compounds.

## References

[1] Su T.C., Yang M.J., Huang H.H., Kuo C.C., Chen L.Y. (2021). Using sensory wheels to characterize consumers’ perception for authentication of Taiwan specialty teas. Foods.

[2] Wang L.F., Lee J.Y., Chung J.O., Baik J.H., So S., Park S.K. (2008). Discrimination of teas with different degrees of fermentation by SPME-GC analysis of the characteristic volatile flavour compounds. Food Chem..

[3] Zhu J.C., Chen F., Wang L.Y., Niu Y.W., Yu D., Shu C., Chen H.X., Wang H.L., Xiao Z.B. (2015). Comparison of aroma-active volatiles in oolong tea infusions using GC-Olfactometry, GC-FPD, and GC-MS. J. Agric. Food Chem..

[4] Greger V., Schieberle P. (2007). Characterization of the key aroma compounds in apricots (*Prunus armeniaca*) by application of the molecular sensory science concept. J. Agric. Food Chem..

[5] Huang W., Fang S., Wang J., Zhuo C., Luo Y., Yu Y., Li L., Wang Y., Deng W.W., Ning J. (2022). Sensomics analysis of the effect of the withering method on the aroma components of Keemun black tea. Food Chem..

[6] Wang J., Li M., Wang H., Huang W., Li F., Wang L., Ho C.T., Zhang Y., Zhang L., Zhai X. (2022). Decoding the specific roasty aroma Wuyi Rock Tea (*Camellia sinensis*: Dahongpao) by the sensomics approach. J. Agric. Food Chem..

[7] (2018). Methodology for Sensory Evaluation of Tea.

[8] Wang D., Liu Z., Chen W., Lan X., Zhan S., Sun Y., Su W., Lin C.C., Ni L. (2023). Comparative study of the volatile fingerprints of roasted and unroasted oolong tea by sensory profiling and HS-SPME-GC-MS. Curr. Res. Food Sci..

[9] Lan X., Liu Z., Wang D., Zhan S., Chen W., Su W., Sun Y., Ni L. (2022). Characterization of volatile composition, aroma-active compounds and phenolic profile of Qingxin oolong tea with different roasting degrees. Food Biosci..

[10] Liu Z., Chen F., Sun J., Ni L. (2022). Dynamic changes of volatile and phenolic components during the whole manufacturing process of Wuyi Rock tea (Rougui). Food Chem..

[11] Yang L., Fan W., Xu Y. (2021). GC × GC-TOF/MS and UPLC-Q-TOF/MS based untargeted metabolomics coupled with physicochemical properties to reveal the characteristics of different type daqus for making soy sauce aroma and flavor type baijiu. LWT-Food Sci. Technol..

[12] Zhu J., Niu Y., Xiao Z. (2021). Characterization of the key aroma compounds in Laoshan green teas by application of odour activity value (OAV), gas chromatography-mass spectrometry-olfactometry (GC-MS-O) and comprehensive two-dimensional gas chromatography mass spectrometry (GC × GC-qMS). Food Chem..

[13] Welke J.E., Manfroi V., Zanus M., Lazarotto M., Alcaraz Zini C. (2012). Characterization of the volatile profile of Brazilian Merlot wines through comprehensive two dimensional gas chromatography time-of-flight mass spectrometric detection. J. Chromatogr. A.

[14] Román-Kustas J., Hoffman J.B., Alonso D., Reed J.H., Gonsalves A.E., Oh J., Hong S., Jo K.D., Dana C.E., Alleyne M. (2020). Analysis of cicada wing surface constituents by comprehensive multidimensional gas chromatography for species differentiation. Microchem. J..

[15] Huang X.H., Zheng X., Chen Z.H., Zhang Y.Y., Du M., Dong X.P., Qin L., Zhu B.W. (2019). Fresh and grilled eel volatile fingerprinting by e-Nose, GC-O, GC-MS and GC × GC-QTOF combined with purge and trap and solvent-assisted flavor evaporation. Food Res. Int..

[16] Sheibani E., Duncan S.E., Kuhn D.D., Dietrich A.M., O’Keefe S.F. (2016). SDE and SPME analysis of flavor compounds in Jin Xuan oolong tea. J. Food Sci..

[17] Radman S., Jerkovic I. (2022). Volatile organic compound profiles of *Cystoseira corniculata* (Turner) zanardini 1841 and *Ericaria amentacea* (C.Agardh) molinari and guiry 2020 (ex. *Cystoseira amentacea* (C.Agardh) bory de saint-vincent, 1832). Molecules.

[18] An Y., Wen L., Li W., Zhang X., Hu Y., Xiong S. (2022). Characterization of warmed-over flavor compounds in surimi gel made from silver carp (*Hypophthalmichthys molitrix*) by gas chromatography-ion mobility spectrometry, aroma extract dilution analysis, aroma recombination, and omission studies. J. Agric. Food Chem..

[19] Kang S., Yan H., Zhu Y., Liu X., Lv H.P., Zhang Y., Dai W.D., Guo L., Tan J.F., Peng Q.H. (2019). Identification and quantification of key odorants in the world’s four most famous black teas. Food Res. Int..

[20] Guo X., Ho C.T., Wan X., Zhu H., Liu Q., Wen Z. (2021). Changes of volatile compounds and odor profiles in Wuyi rock tea during processing. Food Chem..

[21] Guo X., Ho C.T., Schwab W., Wan X. (2021). Effect of the roasting degree on flavor quality of large-leaf yellow tea. Food Chem..

[22] Zhang X., Gao P., Xia W., Jiang Q., Liu S., Xu Y. (2022). Characterization of key aroma compounds in low-salt fermented sour fish by gas chromatography-mass spectrometry, odor activity values, aroma recombination and omission experiments. Food Chem..

[23] Zhan S., Liu Z., Su W., Lin C.-C., Ni L. (2023). Role of roasting in the formation of characteristic aroma of wuyi rock tea. Food Control.

[24] Guo X., Song C., Ho C.T., Wan X. (2018). Contribution of L-theanine to the formation of 2,5-dimethylpyrazine, a key roasted peanutty flavor in Oolong tea during manufacturing processes. Food Chem..

[25] Wang B., Qu F., Wang P., Zhao L., Wang Z., Han Y., Zhang X. (2022). Characterization analysis of flavor compounds in green teas at different drying temperature. LWT-Food Sci. Technol..

[26] Liu X., Liu Y., Li P., Yang J., Wang F., Kim E., Wu Y., He P., Li B., Tu Y. (2021). Chemical characterization of Wuyi rock tea with different roasting degrees and their discrimination based on volatile profiles. RSC Adv..

[27] Song F., Xiang H., Li Z., Li J., Li L., Fang Song C. (2023). Monitoring the baking quality of Tieguanyin via electronic nose combined with GC-MS. Food Res. Int..

[28] Guo X., Schwab W., Ho C.T., Song C., Wan X. (2021). Characterization of the aroma profiles of oolong tea made from three tea cultivars by both GC-MS and GC-IMS. Food Chem..

[29] Wang C., Li J., Wu X., Zhang Y., He Z., Zhang Y., Zhang X., Li Q., Huang J., Liu Z. (2022). Pu-erh tea unique aroma: Volatile components, evaluation methods and metabolic mechanism of key odor-active compounds. Trends Food Sci. Technol..

[30] Fu Y.Q., Wang J.Q., Chen J.X., Wang F., Yin J.F., Zeng L., Shi J., Xu Y.Q. (2020). Effect of baking on the flavor stability of green tea beverages. Food Chem..

[31] Ho C.-T., Zheng X., Li S. (2015). Tea aroma formation. Food Sci. Hum. Well..

[32] Zhang Y., Kang S., Yan H., Xie D., Chen Q., Lv H., Lin Z., Zhu Y. (2022). Insights into Characteristic Volatiles in Wuyi Rock Teas with Different Cultivars by Chemometrics and Gas Chromatography Olfactometry/Mass Spectrometry. Foods.

[33] Zhu Y., Lv H.P., Shao C.Y., Kang S., Zhang Y., Guo L., Dai W.D., Tan J.F., Peng Q.H., Lin Z. (2018). Identification of key odorants responsible for chestnut-like aroma quality of green teas. Food Res. Int..

