# Supplementary material for: Characterization of the Key Aroma Compounds in Dong Ding Oolong Tea by Application of the Sensomics Approach
Source: Foods. 2023 Aug 22;12(17):3158. doi: 10.3390/foods12173158 (PMC10486682; doi:10.3390/foods12173158)
Supplement: Supplementary file 1 [file foods-12-03158-s001.zip › DDT_SI.pdf]

Table S1. Tea samples information.

NO.	Collection locations	Production date	Sampling date	Roasting degree
DDT1	Alishanhutongdi, Taiwan	2021 Spring	2021.6.3	Heavy roasting
DDT2	Alishanzhangshuhu, Taiwan	2021 Spring	2021.6.3	Medium roasting
DDT3	Meishanruifengdayao, Taiwan	2021 Spring	2021.6.3	Medium roasting
DDT4	Lishancuiluan, Taiwan	2021 Spring	2021.6.3	Medium roasting
DDT5	Alishanruili, Taiwan	2021 Spring	2021.6.3	Heavy roasting
DDT6	Alishandayao, Taiwan	2020 Spring	2021.6.3	Medium roasting
DDT7	Alishanjiaoliping, Taiwan	2021 Spring	2021.6.3	Medium roasting
DDT8	Alishanruili, Taiwan	2021 Spring	2021.6.3	Heavy roasting
DDT9	Alishanhaishushan, Taiwan	2021 Spring	2021.6.3	Heavy roasting
DDT10	Alishanhutongdi, Taiwan	2020 Spring	2021.6.3	Medium roasting
DDT11	Alishandayao, Taiwan	2019 Winter	2021.6.3	Medium roasting
DDT12	Cuifeng, Taiwan	2019 Spring	2021.6.3	Heavy roasting
DDT13	Longfengxia, Taiwan	2019 Spring	2021.6.3	Heavy roasting
DDT14	Shanlinxifanzaitian, Taiwan	2021 Spring	2021.6.3	Heavy roasting
DDT15	Luguchuxiang, Taiwan	2021 Spring	2021.6.3	Medium roasting
DDT16	Luguzhangya, Taiwan	2021 Spring	2021.6.3	Medium roasting
DDT17	Zhushan, Taiwan	2021 Spring	2021.6.3	Medium roasting
DDT18	Minjian, Taiwan	2021 Spring	2021.6.3	Medium roasting
DDT19	Nantouminjian, Taiwan	2020 Spring	2021.6.3	Medium roasting
DDT20	Nantouminjian, Taiwan	2020 Spring	2021.6.3	Medium roasting
DDT21	Nantouminjian, Taiwan	2020 Spring	2021.6.3	Medium roasting
DDT22	Nantouminjian, Taiwan	2020 Spring	2021.6.3	Medium roasting
DDT23	Shanlinxi, Taiwan	2020 Spring	2021.6.3	Medium roasting
DDT24	Shanlinxi, Taiwan	2020 Spring	2021.6.3	Medium roasting
DDT25	Songbailing, Taiwan	2020 Spring	2021.6.3	Medium roasting
DDT26	Alishanshizhao, Taiwan	2020 Spring	2021.6.3	Medium roasting
DDT27	Alishan, Taiwan	2020 Winter	2021.6.3	Heavy roasting

Table S2. Sensory scores and sensory grades of DDT.

NO.	Expert 1	Expert 2	Expert 3	Expert 4	Expert 5	Average score	Sensory descriptions	Grade
DDT1	94.5	93	93	92	94	93.3	Caramel	High
DDT2	92	93.5	93	92	92	92.5	Roasted	Medium
DDT3	93	94.5	93	90	90	92.1	Floral	Medium
DDT4	94	92.5	93	90	89	91.7	Caramel	Medium
DDT5	93	93.3	93	93	94	93.3	Caramel	High
DDT6	92	92.0	92	92	92	92.0	Caramel	Medium
DDT7	94	94.5	93	91	92	92.9	Roasted	High
DDT8	95	94	94	94	92	93.8	Floral	High
DDT9	93	94	93	93	94	93.4	Floral	High
DDT10	94	92.5	93	92	92	92.7	Caramel	High
DDT11	94	93.5	92	93	91	92.7	Floral	Medium
DDT12	95	93.5	93	94	94	93.9	Caramel	High
DDT13	96	94	92	95	94	94.2	Fresh	High
DDT14	93	93	92	93	93	92.8	Caramel	High
DDT15	87	86.1	87	86	84.5	86.1	Floral	Low
DDT16	91	93.5	92	91	91	91.7	Floral	Medium
DDT17	91.5	94.5	92	90	89	91.4	Roasted	Low
DDT18	92	92.5	93	92	93	92.5	Roasted	Medium
DDT19	93	92	90	88	90	90.6	Fresh, Floral	Low
DDT20	91	91.0	92	91	90	91.0	Pungent	Low
DDT21	88	89.5	90	89	91	89.5	Lost fresh	Low
DDT22	90	91.5	93	93	90	91.5	Thick	Low
DDT23	87	88.0	86	90	89	88.0	Cloudy	Low
DDT24	92	93	92	92	93	92.4	Caramel	Medium
DDT25	86	84.3	83	85	83	84.3	Floral	Low
DDT26	85	85.3	85	86	85	85.3	Roasted	Low
DDT27	90	92.5	92	91	93	91.7	Caramel	Medium

Table S3. Standard curve of aroma-active compounds of DDT.

NO.	Compounds	Linear equation	Correlation coefficient (R^2)
1	6-Methyl-5-hepten-2-one	y = 14.434x + 0.159	0.9966
2	3-Hexen-1-ol	y = 1.0443x + 0.0166	0.9955
3	*(E)*-Linalool oxide	y = 0.1912x + 0.0417	0.9969
4	2-Ethyl-5-methyl-pyrazine	y = 0.0479x - 0.006	0.991
5	2-Ethyl-3,5-dimethylpyrazine	y = 0.1298x + 0.006	0.9953
6	Furfural	y = 0.1508x + 0.0414	0.9939
7	*(E,E)*-2,4-Heptadienal	y = 0.7624x + 0.0104	0.9962
8	2,3-Diethyl-5-methyl-pyrazine	y = 8.6331x - 0.2272	0.9987
9	2-Acetylfuran	y = 0.5138x + 0.0733	0.9905
10	3,5-Octadien-2-one	y = 0.4476x + 0.1112	0.9912
11	Linalool	y = 1.1166x - 0.0051	0.9967
12	5-Methylfurfural	y = 0.8605x + 0.0518	0.9978
13	Hotrienol	y = 1.1166x - 0.0051	0.9967
14	2-Methyl-4-methoxyaniline	y = 0.0139x + 0.0115	0.9899
15	Caproicacidhexneylester	y = 1.3007x - 0.0632	0.9959
16	Methyl salicylate	y = 0.0684x - 0.0005	0.9915
17	1-Furfurylpyrrole	y = 3.2893x + 0.2565	0.9968
18	Geraniol	y = 33.362x - 0.465	0.9995
19	Phenylethyl alcohol	y = 0.7527x + 0.0371	0.9911
20	*(Z)*-Jasmone	y = 3.0731x - 0.1101	0.9974
21	2-Acetylpyrrole	y = 0.5018x - 0.0385	0.9957
22	*(E)*-Nerolidol	y = 15.108x - 0.7382	0.9912
23	Phenethyl benzoate	y = 1.0847x - 0.0305	0.9975

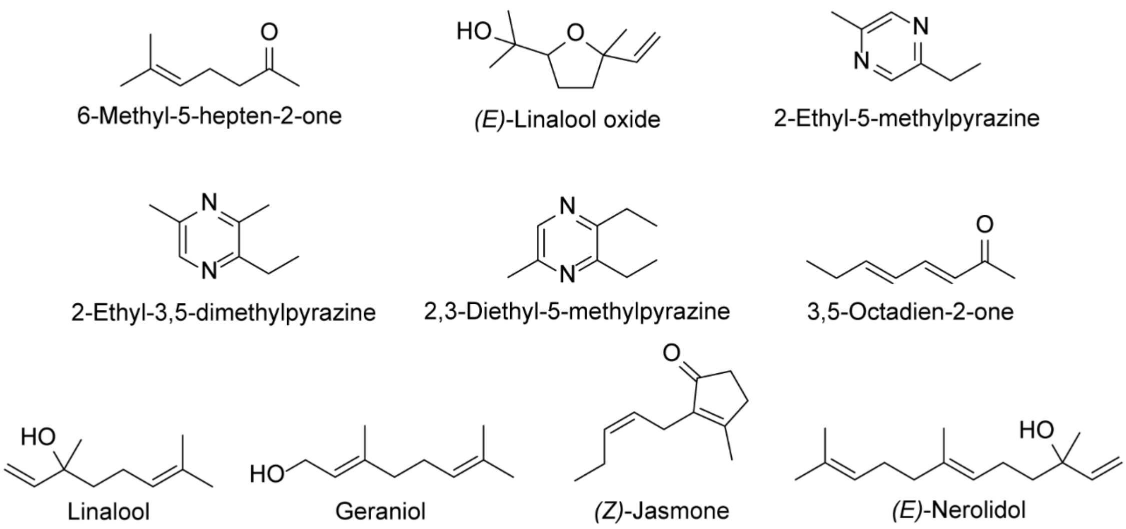

Figure S1. Chemical structure of 10 key aroma compounds.
